# Clonal profile, virulence and resistance of *Staphylococcus aureus* isolated from sheep milk

**DOI:** 10.1590/S1517-838246220131164

**Published:** 2015-06-01

**Authors:** Katheryne Benini Martins, Patricia Yoshida Faccioli-Martins, Danilo Flávio Moraes Riboli, Valéria Cataneli Pereira, Simone Fernandes, Aline A. Oliveira, Ariane Dantas, Luiz Francisco Zafalon, Maria de Lourdes Ribeiro de Souza da Cunha

**Affiliations:** 1 Universidade Estadual Paulista, Departamento de Microbiologia e Imunologia, Instituto de Biociências, Universidade Estadual Paulista, Botucatu, SP, Brasil, Departamento de Microbiologia e Imunologia, Instituto de Biociências, Universidade Estadual Paulista "Júlio de Mesquita Filho", Botucatu, SP, Brazil.; 2 Universidade Estadual Paulista, Departamento de Produção Animal, Faculdade de Medicina Veterinária e Zootecnia, Universidade Estadual Paulista, Botucatu, SP, Brasil, Departamento de Produção Animal, Faculdade de Medicina Veterinária e Zootecnia, Universidade Estadual Paulista "Júlio de Mesquita Filho", Botucatu, SP, Brazil.; 3 Embrapa Pecuária Sudeste, Embrapa Pecuária Sudeste, São Carlos, SP, Brasil, Embrapa Pecuária Sudeste, Empresa Brasileira de Pesquisa Agropecuária, São Carlos, SP, Brazil.

**Keywords:** mastitis, sheep, *Staphylococcus aureus*, antibiotic resistance, virulence factors

## Abstract

The objective of this study was to characterize the clonal profile, virulence factors and antimicrobial resistance, particularly oxacillin resistance, of *Staphylococcus aureus* isolated from sheep milk. Milk samples were collected from all teats for the California Mastitis Test (CMT), somatic cell count, identification of *S. aureus*, investigation in these strains of genes encoding toxins (*sea*, *seb*, *sec*, *sed, tst*), biofilm (*icaA*, *icaC*, *icaD*, *bap*), leukocidin (*luk-PV*) oxacillin resistance by *mecA* gene detection and susceptibility testing (12 antibiotics). Messenger RNA expression was evaluated by RT-PCR in isolates carrying toxin and biofilm genes. Biofilm formation was also evaluated phenotypically by adherence to polystyrene plates. The clonal profile of *S. aureus* was investigated by pulsed-field gel electrophoresis. A total of 473 milk samples were collected from 242 animals on three farms and 20 *S. aureus* strains were isolated and none carried the *mecA* gene. The two *sec* gene-positive isolates and the isolates carrying the *tst* and *luk*-*PV* genes were positive by RT-PCR. *Staphylococcus aureus* isolated from the three flocks studied showed high susceptibility to the drugs tested and none was biofilm producer, indicating that biofilm formation was not a virulence factor causing infection by these strains. The typing of 17 *S. aureus* isolates revealed the presence of a common clone on the three farms studied, and the presence and expression of the *sec* and *tst* genes in one strain of this clone suggest the possible acquisition of virulence genes by this clone, a fact that is important for animal health and food hygiene.

## Introduction

Mastitis is an inflammation of the mammary gland which is characterized by physical, chemical and microbiological alterations in milk and tissue abnormalities. Furthermore, the disease is associated with premature culling of ewes with udder abnormalities and reduced weight gain of lambs. In the study of Moroni *et al.* (2007), the weight gain of lambs nursing dams without mastitis was higher than that of animals nursing ewes with subclinical mastitis. Among the latter, lambs nursing dams with subclinical infection caused by *S. aureus* showed lower weight gain than those nursing dams with mastitis caused by other infectious agents such as coagulase-negative staphylococci, streptococci, and Gram-negative bacteria.

In small ruminants such as sheep, coagulase-negative staphylococci are the microorganisms most frequently isolated from cases of subclinical mastitis (Bergonier *et al.*, 2003; Vautor *et al.*, 2009), whereas *S. aureus* can cause clinical and subclinical mastitis (Contreras *et al.*, 2007). *S. aureus* was the microorganism most frequently isolated from milk of Awassi sheep with subclinical mastitis, accounting for 39% of all isolates, followed by *Streptococcus* spp. (25%), *Escherichia coli* (19.6%), and coagulase-negative staphylococci (17.9%) (Al-Majali and Jawabreh, 2003). Santos *et al.* (2007) studied the clinical aspects and milk characteristics of 10 Santa Inês sheep with clinical mastitis after experimental inoculation of their mammary glands with *S. aureus*. The results showed a reduction in milk production and fat content, in addition to alterations in the physicochemical properties of milk. After treatment, although the animals had recovered from the disease, the function of their mammary glands was completely lost.

Mastitis caused by *S. aureus* can result in long-term infection and can become chronic, with a low rate of cure and consequent loss of milk production (Sabour *et al.*, 2004), since this microorganism possesses different virulence factors that contribute to its persistence in mammary tissue (Santos *et al.*, 2003). Different virulence factors such as enterotoxins, leukocidins and biofilms (Peacock *et al.*, 2002) are important since they contribute to aggravation of the disease and are a matter of public health. Biofilm formation permits adherence and colonization of the mammary gland epithelium (Otto, 2008), leukocidin causes tissues necrosis and leukocyte destruction, and enterotoxins can cause food poisoning (Argudín *et al.*, 2010).

In addition to virulence, a major concern in the control of mastitis is resistance of the etiological agents to antibiotics. Therapeutic success is compromised by the growing number of strains resistant to drugs used indiscriminately in veterinary medicine. Staphylococcal resistance to methicillin is associated with the acquisition of the staphylococcal cassette chromosome *mec* (SCC*mec*), a resistance island consisting of the structural gene, *mecA*. *S. aureus* strains carrying this gene are classified as methicillin (oxacillin)-resistant *S. aureus* (MRSA). These strains are frequently resistant to most antimicrobial agents, including aminoglycosides, macrolides, chloramphenicol, tetracycline, and fluoroquinolones (Wang *et al.*, 2008; Kumar *et al.*, 2010).

Therefore, the objective of the present study was to characterize the clonal profile, virulence factors and antibiotic resistance of *S. aureus* isolated from raw milk of sheep with subclinical mastitis.

## Materials and Methods

### Origin of the isolates

Milk samples were collected from each teat of 242 sheep belonging to the following experimental flocks: 37 from the flock of Instituto de Zootecnia, Nova Odessa, 150 from the flock of Embrapa Pecuária Sudeste, São Carlos, and 55 from the Edgardia Farm, Faculdade de Medicina Veterinária e Zootecnia, Botucatu. All facilities are located in the State of São Paulo, Brazil. The flocks from São Carlos and Nova Odessa were Santa Inês sheep and those from Botucatu were Bergamacia sheep. Milk samples were collected from all animals for microbiological tests for the isolation of *S. aureus*.

Screening for subclinical cases was performed immediately before the collection of milk samples for the microbiological diagnosis of mastitis by the California Mastitis Test (CMT) according to the technique of Schalm and Noorlander (1957). Samples were also collected for somatic cell count (SCC) into flasks containing bronopol for counting in an electronic Somacount 300 (Bentley Instruments^®^). Mammary glands with a positive reaction in the CMT or SCC > 3.0 × 10^5^ cells/mL milk (McDougall *et al.*, 2001) and that were bacteriologically positive were classified as subclinical mastitis.

### Isolation and identification of *S. aureus*

The milk samples were cultured on blood agar at 37 °C for 72 h. Suspected bacterial colonies were stained by the Gram method for inspection of morphology. Colonies characterized as Gram-positive cocci were submitted to catalase and coagulase tests. The genus *Staphylococcus* was differentiated from *Micrococcus* based on the oxidation and fermentation of glucose, resistance to bacitracin (0.04 U), and susceptibility to furazolidone (100 mg). *S. aureus* was identified based on the fermentation of maltose, trehalose, and mannitol (Cunha *et al.*, 2004).

### Antimicrobial susceptibility testing

Twenty *S. aureus* isolates were submitted to *in vitro* susceptibility testing by the disk diffusion method according to the guidelines of the Clinical Laboratory Standard Institute (2012). The following 12 antibiotics were tested: rifampicin (5 μg), linezolid (30 μg), vancomycin (30 μg), clindamycin (2 μg), erythromycin (15 μg), penicillin (10 UI), oxacillin (1 μg), cefoxitin (30 μg), tetracycline (30 μg), gentamicin (10 μg), ciprofloxacin (5 μg), and cotrimoxazole (25 μg).

### DNA extraction and amplification by PCR for detection of the *mecA*, biofilm (*icaADBC* and *bap*) and exotoxin genes (*sea*, *seb*, *sec*, *sed*, *tst and luk-PV*)

Total nucleic acid was extracted from Staphylococcus spp. strains cultured on blood agar, inoculated individually into brain-heart infusion (BHI) broth, and incubated for 24 h at 37 °C. The Illustra^®^ kit (GE Healthcare) was used for extraction. Briefly, staphylococcal cells were first digested with 10 mg/mL lysozyme and 20 mg/mL proteinase K. Next, 500 μL lysis solution was added and the mixture was centrifuged at 5,000 × *g* for 1 min. The supernatant was transferred to a column and centrifuged at 11,000 × *g* for 1 min. The liquid part was discarded and 500 μL lysis solution was again added to the column. After centrifugation at 11,000 × *g* for 1 min and discarding the liquid part, 500 μL washing solution was added and the column was centrifuged at 11,000 × *g* for 3 min. Next, the column was transferred to a 1.5-mL tube and 200 μL Milli-Q water (Millipore, Eschborn, Germany) heated to 70 °C was used for elution. The samples were centrifuged at 5,000 × *g* for 1 min and the column was discarded. The extracted DNA was stored at −20 °C. For detection of the *mecA*, biofilm (*icaADBC*, *bap*) and exotoxin genes (*sea*, *seb*, *sec*, *sed*, *tst and luk-PV*), the primers shown in Table 1 and the parameters recommended by the respective authors were used: Murakami *et al.* (1991), Rohde *et al.* (2007), Arciola *et al.* (2005), Cucarella *et al.* (2001), Johnson *et al.* (1991), and Lina *et al.* (1999).

**Table 1:** Primers used for PCR.

Gene	5′-3′ Nucleotide sequence	Amplification product (bp)	Reference
*icaA*	TGG CTG TAT TAA GCG AAG TC	669	Rohde *et al*., 2007
	CCT CTG TCT GGG CTT GAC C		
*icaC*	TAA CTT TAG GCG CAT ATG TTT	400	Arciola *et al*., 2005
	TTC CAG TTA GGC TGG TAT TG		
*icaD*	ATG GTC AAG CCC AGA CAG AG	198	Arciola *et al*., 2005
	CGTGTTTTCAACATTTAATGCAA		
*icaB*	CTGATCAAGAATTTAAATCACAAA	302	Arciola *et al*., 2005
	AAA GTC CCA TAA GCC TGT TT		
*bap*	CCC TAT ATC GAA GGT GTA GAA TTG CAC	971	Cucarella *et al*., 2001
	GCT GTT GAA GTT AAT ACT GTA CCT GC		
*bap*	CCC TAT ATC GAA GGT GTA GAA TTG CAC	971	Cucarella *et al*., 2001
	GCT GTT GAA GTT AAT ACT GTA CCT GC		
*sea*	TTG GAA ACG GTT AAA ACG AA	120	Johnson *et al*., 1991
	GAA CCT TCC CAT CAA AAA CA		
*seb*	TCG CAT CAA ACT GAC AAA CG	478	Johnson *et al*., 1991
	GCA GGT ACT CTA TAA GTG CC		
*sec*	GAC ATA AAA GCT AGG AAT TT	257	Johnson *et al*., 1991
	AAA TCG GAT TAA CAT TAT CC		
*sed*	CTA GTT TGG TAA TAT CTC CT	317	Johnson *et al.,* 1991
	TAA TGC TAT ATC TTA TAG GG		
*tst*	ATG GCA GCA TCA GCT TGA TA	350	Johnson *et al.*, 1991
	TTT CCA ATA ACC ACC CGT TT		
*luk*-PVL	ATC ATT AGG TAA AAT GTC TGG ACA TGA TCC	433	Lina *et al*., 1999
	GCA TCA ATT GTA TTG GAT AGC AAA AGC		
*mecA*	ATC GAT GGT AAA GGT TGG	533	Murakami *et al*., 1991
	AGT TCT GCA GTA CCG GAT TTG		

The agarose gels were prepared at a concentration of 2% in 1X TBE, stained with SYBR Safe DNA Gel Stain^®^ (Invitrogen), and visualized under a UV transilluminator. Reactions that amplified fragments larger than 1,000 bp were submitted to electrophoresis on 0.8% agarose gel.

### Confirmation of the expression of virulence factors

The expression of the genes encoding enterotoxin A, B, C, D or toxic shock syndrome toxin 1 (TSST-1), biofilm genes (*icaADBC* and *bap*) and *luk-PV* in the *S. aureus* isolates in which these genes were amplified by PCR was confirmed by RT-PCR as described below.

#### RNA extraction

Total RNA was extracted from *S. aureus* cultured on blood agar, inoculated individually into BHI broth, and incubated for 24 h at 37 °C. The Illustra RNAspin Mini RNA kit was used for extraction according to manufacturer instructions. For this purpose, 200 μL of the *S. aureus* culture was transferred to a sterile 1.5-mL Eppendorf tube and centrifuged at 10,000 × *g* for 1 min. Next, the supernatant was discarded, 100 μL TE containing 2 mg/mL lysozyme was added, and the mixture was incubated for 10 min at 37 °C. For cell lysis, 350 μL RA1 buffer was added together with 3.5 μL β-mercaptoethanol. The solution was applied to RNAspin Mini Filter units and centrifuged at 11,000 × *g* for 1 min. The filters were discarded after centrifugation. For adjustment of the binding conditions, 350 μL 70% ethanol was added to the filtrate and the mixture was transferred to an RNAspin Mini Column and centrifuged at 8,000 × *g* for 30 s. For adsorption of RNA to the membrane, 350 μL membrane desalting buffer was added and the mixture was centrifuged at 11,000 × *g* for 1 min. The samples were then washed in two steps. First, 600 μL RA3 buffer was added and the column was centrifuged at 11,000 × *g* for 1 min. For the second wash, 250 μL RA3 buffer was added and the column was centrifuged at 11,000 × *g* for 2 min. The column was then transferred to a new 1.5-mL Eppendorf tube for the elution of RNA. For this purpose, 45 μL RNA-free water containing 5 μL guard RNA was added and the column was centrifuged at 11,000 × *g* for 1 min. DNase treatment for complete elimination of possible DNA residues consisted of the addition of 2 μL buffer and 2 μL DNase and incubation of the mixture for 1 h at 37 °C. Next, 2 μL Stop DNase was added and the mixture was incubated for 10 min at 65 °C for the inhibition of DNase. The extracted RNA was immediately stored at −80 °C.

#### Preparation of cDNA

Two mixtures were prepared (Mix 1 and Mix 2). For Mix 1, 14 μL RNA (divided into aliquots and treated with DNase), 1 μL dNTP, and 4 μL nuclease-free water were used (extraction kit). For Mix 2, 4 μL 5X First-Strand Buffer, 1 μL DTT (0.1 M), and 1 μL SuperScript III (200 U/μL) were used. Mix 1 was incubated in a thermocycler for 5 min at 65 °C, removed from the thermocycler, and immediately put on ice for approximately 5 min. Next, Mix 2 (6 μL) was added and the sample was again placed in the thermocycler and the program was continued with 30 cycles at 65 °C for 5 min, 25 °C for 5 min, 50 °C for 60 min, 70 °C for 15 min, and finished at 20 °C. The cDNA was then frozen at −80 °C.

#### PCR amplification of cDNA

The cDNA obtained was submitted to RT-PCR to determine the expression of toxin and biofilm genes using the primers described in Table 1. The amplified products were visualized by electrophoresis as described in item 2.4.

### Investigation of biofilm formation by adherence to polystyrene plates (Christensen *et al.*, 1985) modified by Oliveira and Cunha (2010)

The method of biofilm formation on culture plates proposed by Christensen *et al.* (1985) and modified by Oliveira and Cunha (2010) was used. This method is based on the spectrophotometric determination of the optical density of the adherent material produced by the bacteria.

Cultures grown in TSB for 24 h and subsequently diluted 1:1 in TSB prepared with 2% glucose were used. The tests were carried out in 96-well flat-bottom plates (Costar, model 3599, Corning). The wells were filled in quadruplicate with 200 μL of the diluted culture. The following international reference strains were included in all tests: *S. aureus* ATCC 29213 (biofilm producer) as positive control and ATCC 33591 (non-producer) as negative control, and *S. epidermidis* ATCC 35983 (biofilm producer) as positive control and ATCC 12228 (non-producer) as negative control, as well as sterile TSB. The plates were incubated for 24 h at 37 °C. After this period, the content of each well was carefully aspirated with a multichannel pipette and the wells were washed four times with 200 μL phosphate-buffer saline, pH 7.2. The plates were dried at room temperature for 1 h. Next, the wells were stained with 2% crystal violet for one minute, the volume was aspirated, and excess dye was removed by washing the plates with distilled water using a multichannel pipette. The plates were then dried at room temperature for 60 min and optical density was read in a Labsystem Multiskan EX microplate reader equipped with a 540-nm filter. The strains were classified as negative when the cut-off value corresponded to the classification of non-adherent, and as positive when the cut-off value corresponded to the classification of weakly or strongly adherent.

### Pulsed-field gel electrophoresis (PFGE)

PFGE of the *S. aureus* isolates was done according to a modification of the protocol of McDougal *et al.* (2003). In a previously weighed microtube, 0.5 mL of an overnight culture of *S. aureus* was centrifuged at 12,000 rpm for 50 s. After discarding the supernatant, the microtube was weighed again and 300 μL TE (10 mM Tris, 1 mM EDTA, pH 8.0) plus the difference between the final and initial weight in mL were added. The samples were left to stand in a water bath for 10 min at 37 °C. After homogenization, 5 μL lysostaphin (1 mg/mL in 20 mM sodium acetate, pH 4.5) and 300 μL low-melting agarose were added. The mixtures were poured into plug molds and the plugs were allowed to solidify. The plugs were then placed in 2 mL EC solution (6 mM Tris-HCl, 1 M NaCl, 100 mM EDTA, 0.5% Brij-58, 0.2% sodium deoxycholate, 0.5% sodium lauryl sarcosinate) and incubated at 37 °C for at least 4 h. The EC solution was discarded and the plugs were washed four times with 2 mL TE for 30 min at room temperature.

Genomic DNA was restricted with *Sma*I (Fast Digest SmaI^®^, Fermentas Life Science, Canada) in 50 μL restriction buffer using half the plug. Electrophoresis was carried out in a CHEF-DR III System^®^ (BioRad Laboratories, USA) using 1% agarose gel (Pulsed Field Certified Agarose, BioRad Laboratories, USA) prepared in 0.5X TBE under the following running conditions: pulse time of 5 to 40 s for 21 h; linear ramp; 6 V/cm; angle of 120°; 14°C; 0.5X TBE as running buffer. The Lambda Ladder PFG Marker^®^ (New England BioLabs) was used as molecular marker. The gels were stained with GelRed^®^ (10,000X in water, Biotium, USA) for 45 min and photographed under UV transillumination. For analysis of similarity, the Dice correlation coefficient was calculated and a dendrogram was constructed by the UPGMA method (unweighted pair group method using arithmetic averages) using the BioNumerics^®^ software (version 6.1; Applied Maths, Belgium).

## Results

Microorganisms were detected in 169 (35.7%) of the 473 milk samples collected from the 242 animals included in the study. Twenty (11.8%) of the microorganisms isolated were identified as *S. aureus*, including 18 (90%) strains isolated from cases of subclinical mastitis and only two (10%) from animals without mastitis. Four of the 20 strains were isolated in the flock from Botucatu, two in the flock from Nova Odessa, and 14 in the flock from São Carlos.

### Antimicrobial resistance

The *mecA* gene was not detected in any of the *S. aureus* strains and most isolates were susceptible to all antimicrobial agents tested, except for one strain that was resistant to tetracycline.

### Toxin detection

Seven (35%) of the *S. aureus* isolates carried one or more exotoxin genes (Table 2). Three (15%) of these seven strains that tested positive by PCR for exotoxin genes were positive by RT-PCR. One isolate carrying the *sec*+*tst* genes expressed the two genes concomitantly, one isolate carrying the *sea*+*seb*+*luk-PV* genes expressed only the luk-PV gene, and the third isolate positive for the *sea*+*sec* genes expressed only the *sec* gene.

**Table 2:** Detection and expression of exotoxin genes.

Gene/expression	PCR N (%)	RT-PCR N (%)
sea+*seb*+*luk-PV/*PVL	1 (5.0)	1 (5.0)
*sea+seb*	2 (10.0)	0
*sec*+tst/SEC+TSST-1	1 (5.0)	1 (5.0)
*sea+sec/*SEC	1 (5.0)	1 (5.0)
Total	7 (35.0)	3 (15.0)

*sea*, *seb*, *sec*, *tst*, *luk-pv*: presence of the genes encoding toxins A, B, C, TSST-1, and Panton-Valentine leukocidin (PVL). PVL, TSST-1 and SEC: Toxin expression. N: number of isolates; %: percentage of isolates.

### Biofilm detection

With respect to biofilm genes (Table 3), the complete *icaADBC* operon was detected in one of the isolates. The *icaA* + *icaD* + *icaB* were concomitantly present in five isolates, *icaA + icaD* in six isolates, three isolates only carried the *icaD* gene, and one isolate only carried the *icaB* gene. The *bap* gene was detected in four (20%) isolates, but none of the strains expressed this gene.

**Table 3:** Detection and expression of biofilm genes.

Genes	PCR N (%)	RT-PCRN (%)
*icaADBC*	1 (5.0)	0
*icaA+ icaD + icaB*	5 (25.0)	0
*icaA + icaD*	3 (15.0)	0
*icaA + icaD + bap*	3 (15.0)	0
*icaD+bap*	1 (5.0)	0
*icaD*	3 (15.0)	0
*icaB*	1 (5.0)	0
Total	17 (85.0)	0

*icaA*, *icaD*, *icaB*, *icaC*, *bap*: biofilm genes; N: number of isolates; %: percentage of isolates.

None of the isolates exhibited a positive result in the phenotypic test of biofilm formation on polystyrene plates.

### Typing

**Figure 1:**
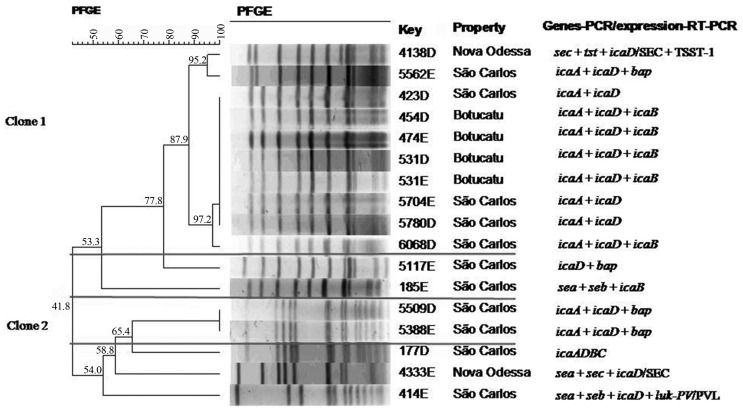
Dendrogram generated by Dice analysis/UPGMA (Bionumerics, Applied Maths) of the *Sma*I PFGE profiles of 17 *S. aureus* isolates (similarity ≥ 80%).

Molecular typing by PFGE was performed on only 17 strains since three isolates from the São Carlos flock could not be typed. PFGE (Figure 1) revealed the presence of two clones, a larger one (clone 1) comprising 10 isolates (one from Nova Odessa, five from São Carlos, and four from Botucatu) and a smaller one (clone 2) comprising only two isolates from São Carlos. The remaining five isolates (one from Nova Odessa and four from São Carlos) exhibited a polyclonal profile.

With respect to virulence factors (Table 4), in clone 1 (10 isolates) only one strain carried an enterotoxin gene and the TSST-1 gene and expressed the two genes. The *bap* gene was detected in one isolate and the *icaB* gene of the *icaADBC* operon was detected in five isolates, but no expression of these genes related to biofilm formation was observed.

**Table 4:** Characteristics of the *S. aureus* clones isolated from sheep milk samples.

Clone	Toxin gene	Toxin expression	Biofilm genes	Biofilm expression
1 (10 isolates)	*sea* (0)	SEA (0)	*icaA* (9)	*icaA* (0)
	*seb* (0)	SEB (0)	*icaD* (10)	*icaD* (0)
	*sec* (1)	SEC (1)	*icaB* (5)	*icaB* (0)
	*sed* (0)	SED (0)	*icaC* (0)	*icaC* (0)
	*tst* (1)	TSST-1 (1)	*bap* (1)	*bap* (0)
	*luk*-PV (0)	PVL (0)		
2 (2 isolates)	*sea* (0)	SEA (0)	*icaA* (2)	*icaA* (0)
	*seb* (0)	SEB (0)	*icaD* (2)	*icaD* (0)
	*sec* (0)	SEC (0)	*icaB* (0)	*icaB* (0)
	*sed* (0)	SED (0)	*icaC* (0)	*icaC* (0)
	*tst* (0)	TSST-1 (0)	*bap* (2)	*bap* (0)
	*luk*-PV (0)	PVL (0)		

None of the toxin genes studied was detected in clone 2 (two isolates). Regarding the genes for biofilm formation, only the *bap* gene was detected in two isolates by PCR, but not by RT-PCR. The genes of the *icaADBC* operon were not detected in any of the isolates by RT-PCR.

With respect to the virulence of *S. aureus* isolated from different flocks, it is important to highlight the detection of the *sea* + *sec* and *sec + tst* genes in two strains isolated from the farm in Nova Odessa, with the expression of SEC and TSST-1 being confirmed by RT-PCR (Figure 1). In the flock from São Carlos, the *luk*-*PV* gene was detected and expressed in one isolate. Additionally, four *bap* gene-positive isolates were from this farm and one isolate carried the complete *icaADBC* operon. No toxigenic *S. aureus* strains were isolated from the farm in Botucatu; however, the *icaADC* genes were detected in four strains isolated from animals of this flock.

## Discussion

*S. aureus* is a versatile microorganism that causes infection in different hosts. Moreover, this bacterium is one of the most important pathogens in the etiology of infectious mastitis in cows, goats, and sheep, causing chronic infection of the mammary tissue that is difficult to treat (Aires-de-Souza *et al.*, 2007). In the present study, *S. aureus* was identified in 20 (11.8%) milk samples collected from sheep with subclinical mastitis.

The *mecA* gene was not detected in any of the *S. aureus* strains studied. Vyletelová *et al.* (2011) also did not find the *mecA* gene in *S. aureus* strains isolated from sheep milk, whereas the gene was detected in 20 (6.1%) *S. aureus* strains isolated from cow milk. According to Zafalon *et al.* (2012), *S. aureus* strains that carry oxacillin resistance mediated by the *mecA* gene are generally more resistant to other antibiotics than oxacillin-sensitive microorganisms. In the present study, antimicrobial susceptibility testing revealed only one *S. aureus* isolate that was resistant to tetracycline. The remaining isolates were susceptibility to all drugs tested. Similar results have been reported by Vyletelová *et al.* (2011) for *S. aureus* isolates from sheep. Pengov and Ceru (2003), investigating *S. aureus* isolates obtained from sheep milk samples, demonstrated high rates of susceptibility to the drugs tested. A resistance rate of 6.3% was only observed for penicillin and ampicillin.

Intramammary infections caused by *S. aureus* can have severe consequences for human health because of the production of toxins by this microorganism. These toxins are secreted and remain stable in milk, causing food poisoning (Balaban and Rasooly, 2000; Fagundes and Oliveira, 2004). In the present study, seven isolates carried at least one enterotoxin gene or the TSST-1 gene, with the *seb* gene being the most frequent enterotoxin followed by the *sea* and *sec* genes. The *sed* gene was not detected in any of the isolates. In contrast, the *sec* gene was the most frequent in the study of Scherrer *et al.* (2004). The authors detected this gene in 123 (64.4%) of 191 *S. aureus* strains isolated from milk samples of sheep and goats, followed by *seg* (16.2%), *sea* (14.6%), *sej* (13.6%), *sei* (12.6%), *seb* (2.1%), and *sed* (2.1%). In the literature, *sea* and *seg* (Scherrer *et al.*, 2004; Wang *et al.*, 2009) and *sec*, *sed*, *seg* and *sei* (Zecconi *et al.*, 2006) are the genes most frequently detected in *S. aureus* isolated from animals.

Although the *seb* gene was the most frequent, it was not expressed in any of the *S. aureus* isolates. Only two (10%) isolates expressed the *sec* gene and one (5%) the *tst* gene. Enterotoxins A and D are the toxins most frequently implicated in outbreaks of food poisoning (Ertas *et al.*, 2010). However, the present results demonstrate the importance of enterotoxin C and TSST-1 in strains isolated from sheep. Enterotoxin C has been frequently detected in cases of clinical mastitis in cattle, goats and sheep (Jones and Wieneke, 1986). In England, Bone *et al.* (1989) reported cases of food poisoning caused by the consumption of sheep milk and cheese. Analysis of the cheese samples showed the presence of enterotoxins. Further analysis of milk and cheese samples led the authors to conclude that food contamination with *S. aureus* did not occur during production, but was the result of infection of the milk-producing animals.

The exotoxin Panton-Valentine leukocidin (PVL) was detected in only one strain and its expression was confirmed. PVL is one of the most important virulence factors produced by *S. aureus*, contributing to the pathogenicity of this microorganism. This toxin is associated with different diseases in humans, such as pneumonia and necrotizing dermatitis (Giudice *et al.*, 2009). The *luk-PV* gene has also been identified in *S. aureus* strains isolated from cases of mastitis (Zecconi *et al.*, 2006; Aires-de-Souza *et al.*, 2007; Unal *et al.*, 2012). However, this is the first report showing the expression of this toxin in *S. aureus* isolated from sheep.

Among the different *S. aureus* virulence factors studied, biofilm formation in isolates obtained from cases of sheep mastitis is a poorly investigated aspect. The *icaADBC* operon is responsible for the synthesis of polysaccharide intercellular adhesin (PIA), the main component of the staphylococcal biofilm (Otto, 2008). In the present study, only one isolate carried the complete *icaADBC* operon, whereas other genes of the operon, mainly *icaA* + *icaD*, were detected in most isolates (Table 3). However, none of the isolates expressed the *ica* genes detected. The *bap* gene encodes a protein that plays an important PIA-independent role in biofilm formation (Cucarella *et al.*, 2004). This gene was detected in four strains (Table 4). However, RT-PCR showed no expression of this gene in any of the *S. aureus* isolates. The *bap* gene is usually not detected and its presence has only been reported in a few *S. aureus* strains isolated from cases of bovine subclinical mastitis (Cucarella *et al.*, 2001). The present results differ from those reported by Tel *et al.* (2012) who analyzed 110 *S. aureus* strains isolated from cases of sheep clinical mastitis. The isolates did not carry the *bap* gene, but were positive for the *icaA* and *icaD* genes. According to Aguilar *et al.* (2001), most *S. aureus* strains isolated from mastitis cases are surrounded by a biofilm layer that facilitates adhesion and colonization of the mammary gland epithelium. However, the 20 isolates from sheep studied here did not express the genes studied (*icaADBC* and *bap*).

Typing of 17 *S. aureus* strains revealed the presence of a common clone on the three farms studied. The characterization of the genetic diversity of *S. aureus* is important to understand the pattern of dispersion of the pathogen. The results showed no major heterogeneity among *S. aureus* strains, with half the isolates belonging to a single clone. With respect to the virulence profile of these strains, only one expressed the *sec* and *tst* genes, while the other strains expressed none of the factors studied. These findings suggest that other virulence factors are related to the capacity of this clone to spread among flocks and to successfully establish an infection in the mammary gland of sheep, causing mastitis. The *sec* and *tst* genes are located on a plasmid and pathogenicity island, respectively, and can be transferred from one bacterium to another. The presence of these genes in one strain suggests that this clone, which is able to spread among the flocks studied, probably acquired these virulence genes from other staphylococci, rendering it more virulent.

## Conclusion

Most of the *S. aureus* strains identified were isolated from cases of subclinical mastitis. Only two isolates did not cause infection, a finding demonstrating the importance of *S. aureus* in the etiology of sheep mastitis. All *S. aureus* strains isolated from the three flocks showed high susceptibility to the drugs tested. None of the isolates was a biofilm producer, indicating that biofilm formation was not a virulence factor causing infection by these strains. However, a toxigenic potential was demonstrated for some of the isolates, which expressed SEC, TSST-1 and PVL. A clone that had spread among sheep flocks was identified among the *S. aureus* strains isolated from sheep milk. In addition, the presence and expression of the *sec* and *tst* genes in one strain of this clone suggest the possible acquisition of virulence genes by this clone, a fact that is important for animal health and food hygiene.
